# Increasing baseline aortic valve peak flow velocity is associated with progression of aortic valve stenosis in osteoporosis patients—a possible link to low vitamin D status

**DOI:** 10.1007/s11657-023-01339-2

**Published:** 2023-10-24

**Authors:** Toshihiro Tsuruda, Taro Funamoto, Chiyoko Suzuki, Yoshimasa Yamamura, Michikazu Nakai, Etsuo Chosa, Koichi Kaikita

**Affiliations:** 1 Cardiorenal Research Laboratory, Department of Vascular Advanced Medicine, Faculty of Medicine, University of Miyazaki, 5200 Kihara, Kiyotake, Miyazaki, 889-1692 Japan; 2 Division of Internal Medicine, Cardiovascular Medicine and Nephrology, Faculty of Medicine, University of Miyazaki, 5200 Kihara, Kiyotake, Miyazaki, 889-1692 Japan; 3 Division of Orthopaedic Surgery, Department of Medicine of Sensory and Motor Organs, Faculty of Medicine, University of Miyazaki, 5200 Kihara, Kiyotake, Miyazaki, 889-1692 Japan; 4 Clinical Laboratory, University of Miyazaki Hospital, Faculty of Medicine, University of Miyazaki, 5200 Kihara, Kiyotake, Miyazaki, 889-1692 Japan; 5 Clinical Research Support Center, University of Miyazaki Hospital, Faculty of Medicine, University of Miyazaki, 5200 Kihara, Kiyotake, Miyazaki, 889-1692 Japan

**Keywords:** Osteoporosis, Valvular disease, Calcium, Vitamin D, Echocardiogram

## Abstract

**Purpose:**

The purpose of this study was to investigate the morphological characteristics of the aortic valve and identify factors associated with the progression of aortic valve stenosis (AS) in osteoporosis patients.

**Methods:**

In this single-center prospective cohort study, we recruited 10 patients (mean age: 75 ± 7 years, 90% female) who were taking anti-resorptive medicines at the outpatient clinic of University of Miyazaki Hospital, Japan. Baseline assessments, including transthoracic echocardiogram, blood sampling, and dual energy X-ray absorptiometry, were performed. Follow-up assessments were conducted at 6, 12, 18, and 24 months.

**Results:**

During the 2-year follow-up, three patients with aortic valve peak flow velocity (AV PFV) ≥2 m/s at baseline developed moderate AS, which is defined as AV PFV ≥3 m/s. However, seven patients with AV PFV <2 m/s did not exhibit any progression of AS. There were significant variations in terms of bone mineral density, T-score values, and biomarkers associated with bone turnover (i.e., bone alkaline phosphatase, tartrate-resistance acid phosphatase-5b) among the enrolled patients, but none of these factors were found to be associated with the progression of AS. All patients exhibited low vitamin D status, with a median level of 16.1 ng/mL (25^th^ percentile, 9.7 ng/mL; 75^th^ percentile, 23 ng/mL). The baseline levels of AV PFV values were negatively correlated with 25-hydroxyvitamin D levels, determined by univariate linear regression analysis (beta coefficient = -0.756, 95% confidence interval, -0.136 – -0.023, p = 0.011).

**Conclusion:**

Our data suggest that low vitamin D status might be a potential risk factor for the progression of AS in osteoporosis patients undergoing treatment with anti-resorptive medicines.

***Summary*:**

Elderly patients with osteoporosis patients exhibited a subset of aortic valve stenosis. Our data suggest that the baseline aortic valve peak flow velocity predicts the progression of aortic valve stenosis, and there might be an association between the progression and the co-existing low vitamin D status in these patients.

**Supplementary Information:**

The online version contains supplementary material available at 10.1007/s11657-023-01339-2.

## Introduction

Degenerative calcified aortic valve stenosis (AS) is the most prevalent form of valvular heart disease [1]. Its prevalence in the elderly population (>75 years) is reported to be 12.4% [1], and it increases with age, with an average prevalence rate of 0.2% in the 50–59-old cohort and 9.8% in the 80–89-year-old cohort [2]. The pathogenesis of AS suggests an active biological process characterized by the deposition of lipid into the intima cusps and subsequent lipid oxidation, which triggers an inflammatory response and exacerbates oxidative stress. This sets in motion a self-perpetuating cycle leading to progressive valve calcification [3–5].

Mild-to-moderate and severe AS have been incidentally observed in 26% (142/550) [6] and 3.2% (73/2274) [7] of patients with osteoporotic hip fractures, respectively. The imbalance between bone resorption and formation has been suggested to play a role in AS progression [8–10]. However, the effectiveness of pharmacological interventions targeting the osteogenic pathways in the time course of AS is yet to be proven [4, 11–14]. Low vitamin D status may have multiple adverse health consequences, such as skeletal fragility, muscle weakness, and effect on non-skeletal morbidities, including cardiovascular diseases [15, 16]. Based on this, we hypothesized that the progression of AS is associated with bone and mineral metabolism, and it may be influenced by the individual’s vitamin D status. We conducted a pilot study to investigate the temporal changes in aortic valve morphology and parameters related to bone and mineral metabolism. Additionally, our objective was to determine the factors associated with the progression of AS in osteoporosis patients who were receiving anti-resorptive medicines.

## Methods

### Study protocol

In this single-center prospective cohort study conducted at the outpatient clinic of University of Miyazaki Hospital, Japan between November 20, 2017, and March 31, 2020, the inclusion criteria were as follows: i) Japanese osteoporosis patients regardless of etiology, ii) age ≥50 years for both sexes, and iii) patients who were already taking or had recently started anti-resorptive therapy (bisphosphonate or denosumab). The exclusion criteria were: i) scheduled aortic valve replacement within 6 months, and ii) undergoing regular hemodialysis for end-stage renal disease. A total of 10 patients with osteoporosis were recruited by an orthopedist (T.F.), who was unaware of any prior diagnosis of valvular heart disease at the time of enrollment. Clinical history and medication details were obtained from the patients’ medical records. All patients underwent echocardiography, blood sampling, and dual energy X-ray absorptiometry (DXA) at baseline and at 6, 12, 18, and 24 months of follow-up. Hypertension was defined as having a systolic blood pressure of ≥140 mmHg, a diastolic blood pressure of ≥90 mmHg, or taking any antihypertensive medications. Diabetes mellitus was defined as having a fasting plasma glucose level of ≥126 mg/dL, a 2-hour postprandial glucose of ≥200 mg/dL, or taking any medication for diabetes mellitus. Dyslipidemia was defined as having a fasting plasma total cholesterol level of ≥220 mg/dL and/or triglyceride level of ≥150 mg/dL, or taking any medication for dyslipidemia. Blood pressure measurements were taken with the patient in a seated position at the hospital. Primary osteoporosis was diagnosed when no other disease-causing low mineral density, apart from osteoporosis, was observed, and the results of bone assessment met the required criteria [17]. Secondary osteoporosis was defined as the presence of low bone mass with accompanying endocrine disorders, such as hyperthyroidism, hyperparathyroidism, or diabetes mellitus; rheumatoid arthritis; or drug-induced osteoporosis caused by medications such as glucocorticoids, thyroid hormones, or hypogonadism-inducing agents [18]. The orthopedist (T.F.) routinely adjusted the type and dosage of osteoporosis drugs (Online Resource 1).

### Laboratory measurements

Serum albumin, calcium, phosphate, and creatinine levels were immediately measured using a fully automated clinical chemistry analyzer (TBA-2000FR, Canon Medical Systems Corporation, Tochigi, Japan). For further analysis, serum/plasma was separated by centrifugation at 3,000 rpm for 20 minutes, frozen at −80 °C, and stored on-site without undergoing a freeze–thaw cycle. The following biomarkers were measured: brain natriuretic peptide (BNP) (CLIA, Abbott), which serves as a marker of heart failure; bone alkaline phosphatase (BAP) (CLEIA, Beckman Coulter, Inc), a marker of bone formation; tartrate resistance acid phosphatase (TRACP)-5b (EIA, NITTO BOSEKI Co., Ltd), a marker of bone resorption; fibroblast growth factor (FGF)-23 (C-terminal, Sandwich ELISA, Cat. No. Bl-20702, BIOMEDICA); intact parathyroid hormone (iPTH) (ECLIA, Roche Diagnostic); osteoprotegerin (OPG) (ELISA, Cat. No. K1011, Immunodiagnostik AG); receptor activator of nuclear factor kappa B ligand (RANKL) (free soluble, Sandwich ELISA, Cat. No. Bl-20462, BIOMEDICA); 25-hydroxyvitamin D [25(OH)D, Sandwich ELISA, Fujirebio Inc.]; and 1,25-dihydroxyvitamin D [1,25(OH)_2_D, radioimmunoassay, Immunodiagnostic Systems, Ltd.]. The estimated glomerular filtration rate (eGFR) was calculated using the following formula: eGFR (mL/min/1.73 m^2^) = 194 × (serum Cr)^−1.094^ × (age)^−0.287^ (× 0.739, when female) [19].

### Echocardiography

All study echocardiograms were performed by dedicated ultra-sonographers using the following equipment: Aplio 400 (Canon Medical Systems, Tochigi, Japan), EPIQ7G (Philips Healthcare, Amsterdam, Netherland), and iE33 (Philips Healthcare, Amsterdam, Netherland). A standardized protocol, in accordance with international guidelines [20, 21], was used for the echocardiographic examinations. The left ventricular mass index was measured and indexed to the body surface area using the equation provided by Devereux et al. [22]. The antegrade systolic velocity across the aortic valve was measured using continuous-wave Doppler ultrasound [20]. The aortic valve area was assessed on two-dimensional echocardiograms using the continuity equation. The mean pressure gradient was calculated using pulse and continuous-wave Doppler assessment of the aortic valve. AS severity was categorized based on standard definitions for peak velocity (mild: 2.0–2.9 m/s, moderate: 3.0–3.9 m/s, severe: ≥4.0 m/s) or mean gradient (mild: <20 mm Hg, moderate: 20–39 mm Hg, severe: ≥40 mm Hg) [23].

### DXA

DXA is the standard technique employed in clinical practice for measuring bone mineral density (BMD) in units of g/cm^2^, which is crucial for assessing fracture risk. Scans were obtained for the posterior–anterior lumbar spine (L2–L4) and hip, following the standard practice in Japan [24–26] and the guidelines [17]. BMD measurements were conducted using the Discovery A DXA system (Hologic, MA, USA). T-scores were calculated using young Japanese females as the reference population [17].

### Endpoints

The primary endpoint of this study was to evaluate the morphological changes of the aortic valve, as assessed by AV PFV, mean pressure gradient, and aortic valve area index at the 24-month follow-up. The progression of AS was defined as a change in AS severity to moderate AS, indicated by a peak velocity greater than 3.0 m/s and a mean gradient higher than 20 mmHg. In cases where a patient did not attend the 24-month visit, either the 12- or 18- month visit was used as the final visit for analysis. The secondary endpoint aimed to explore the parameters associated with the progression of AS.

### Statistical analyses

All statistical analyses were performed using SPSS software, version 29 (IBM Corp., Armonk, NY, USA). Data were presented as means ± standard deviations for continuous variables, and as medians with interquartile range (Q1 – Q3). Categorical variables were expressed as the number of patients. Spaghetti graphs were used to visualize the measurements of baseline, 6-, 12-, 18-, and 24-month follow-up for each patient. Furthermore, univariate linear regression analysis was conducted to examine the association between 25(OH)D levels and AV PFV values at baseline. The coefficient (β) and 95% confidence interval (CI) were calculated. A p-value <0.05 was statistically significant.

## Results

### Patients’ characteristics

The baseline characteristics of the patients are presented in Table 1. Among the ten patients, nine were menopausal women. Eight patients had comorbidities, with hypertension being the most prevalent disorder (6/10, 60%). Eight patients were using bisphosphonates, while two were using denosumab as anti-resorptive therapy. At baseline, seven patients were using activated vitamin D_3_ analogs (6 with eldecalcitol and 1 with alfacalcidol) in combination with bisphosphonates (see Online Resource 1). Two patients received native vitamin D and calcium supplement when administered denosumab [27]. Daily calcium intake was not assessed. The laboratory and DXA results at baseline are listed in Table 2. All patients exhibited low vitamin D status, with plasma 25(OH)D levels below 30 ng/mL, and 6 of them had levels below 20 ng/mL. Four patients had a T-score of ≤-2.5 at the lumbar spine, two at the total hip, and two at the femoral neck. The main echocardiographic values at baseline are presented in Table 3. All patients had a tricuspid aortic valve, and mitral valve calcification was not observed in any patient. The left ventricular ejection fraction was preserved.

**Table 1:** Characteristics of patients enrolled in this study

Characteristic	Mean ± SD, or absolute number
Age	75±7
Sex (Male / Female)	1 / 9
Body mass index (kg/m^2^)	22±3
Menopause	9
Current cigarette smoking	0
Alcohol drinking	
(daily / sometime / never)	2 / 2 / 6
Blood pressure (mmHg)	
Systole	135±17
Diastole	81±11
Physical activity	
5-item frailty screening index (0 / 1 / 2 / 3)	5 / 4 / 0 / 1
Osteoporosis	
Primary	8
Endocrine disorders	1
Glucocorticoid-induced	1
Personal history of fracture	4
Comorbidity	
Hypertension	6
Diabetes Mellitus	0
Dyslipidemia	1
Thyroid disease	2
Renal disease	1
Cardiovascular disease (CAD/VHD)	3 (2/1)
Medications	
Osteoporosis	
Bisphosphonates	8
Denosumab	2
Calcium · Vitamin D supplement	2
Activated vitamin D_3_ analog (eldecalcitol : alfacalcidol)	7 (6 : 1)
Vitamin K_2_	1
Cardiovascular	
Calcium channel blocker	5
Angiotensin II receptor blocker	1
Metabolism	
Statin	2
Digestive	
Proton pump inhibitor	2

Data are expressed as mean ± SD (standard deviation) (n = 10), or absolute number. The FRAIL scale includes five components, including Fatigue, Resistance, Ambulation, Illness, and Loss of weight, which range from 0 to 5 (i.e., 1 point for each component; 0 = best to 5 = worst) and represent frail (3–5), pre-frail (1–2), and robust (0) health status. CAD, coronary artery disease; VHD, valvular heart disease

**Table 2:** Laboratory data and dual energy X-ray absorptiometry at baseline

	Mean ± SD	Median (Q1, Q3)
Hemoglobin (g/dL)	12 ± 1	12 (11, 13)
Albumin (g/dL)	4.0 ± 0.3	4.0 (3.8, 4.3)
Creatinine (mg/dL)	0.65 ± 0.10	0.67 (0.54, 0.75)
eGFR (mL/min/1.73 m^2^)	71 ± 19	64 (56, 90)
Albumin-corrected Calcium (mg/dL)	9.2± 0.3	9.2 (8.9, 9.5)
Phosphate (mg/dL)	3.5 ± 0.4	3.5 (3.2, 3.7)
LD (U/L)	195 ± 35	187 (176, 215)
CRP (mg/dL)	0.09 ± 0.12	0.05 (0.02, 0.10)
BNP (pg/mL)	39 ± 52	26 (12, 35)
BAP (µg/L)	8.6 ± 5.0	6.6 (5.4, 9.8)
TRACP-5b (mU/dL)	327 ± 182	286 (157, 474)
FGF-23 (pmol/L)	1.07 ± 1.00	0.62 (0.21, 1.99)
iPTH (pg/mL)	49 ± 22	46 (36, 60)
RANKL (pmol/L)	0.30 ± 0.04	0.30 (0.26, 0.32)
OPG (pmol/L)	5.8 ±1.8	5.9 (4.8, 7.0)
25(OH)D (ng/mL)	16.4± 6.4	16.1 (9.7, 23)
1,25(OH)_2_D (pg/mL)	75.8± 40.7	66.9 (45.1, 92.0)
BMD (g/cm^2^)		
Lumbar spine (L2-L4)	0.78 ± 0.15	0.75 (0.67, 0.87)
Total hip	0.68 ± 0.08	0.64 (0.61, 0.75)
Femoral neck	0.56 ± 0.06	0.56 (0.53, 0.61)
T-score		
Lumbar spine (L2-L4)	-2.05 ± 1.24	-2.20 (-3.00, -1.73)
Total hip	-1.72 ± 0.79	-2.00 (-2.26, -0.98)
Femoral neck	-2.06 ± 0.55	-2.25 (-2.38, -1.58)

Data are expressed as mean ± SD (standard deviation) and median (Q1, Q3) (n = 10, except for 1,25(OH)_2_D, n = 9). Q1 = 25%, Q3 = 75% quantile. BMD, bone mineral density; eGFR, estimated glomerular filtration rate; LD, lactate dehydrogenase; BAP, bone alkaline phosphatase; TRACP-5b, tartrate-resistant acid phosphatase-5b; FGF-23, fibroblast growth factor-23; iPTH, intact parathyroid hormone; RANKL, receptor activator nuclear kappaB ligand; OPG, osteoprotegerin; 25(OH)D, 25-hydroxyvitamin D; 1,25(OH)_2_D, 1,25-dihydroxyvitamin D; T-score, the number of standard deviations above or below the mean for the reference population of young Japanese females (<−2.5 SD, indicating osteoporosis; −1 to −2.5, indicating osteopenia; >−1, indicating normal bone density).

**Table 3:** Echocardiographic features at baseline

	Mean ± SD	Median (Q1, Q3)
LVEF (%)	66 ± 2	66 (64, 68)
IVSTd (mm)	9 ± 2	9 (8, 11)
LVPWTd (mm)	9 ± 2	9 (8, 11)
LVIDd (mm)	42 ± 4	41 (38, 44)
LVIDs (mm)	26 ± 4	26 (21, 28)
LVMI (g/m^2^ body surface area)	89 ± 21	85 (74, 101)
AV PFV (m/s)	1.8 ± 0.7	1.5 (1.3, 2.4)
AVAI (cm^2^/m^2^ body surface area)	1.4 ± 0.3	1.5 (1.1, 1.6)
Mean PG (mmHg)	7.6 ± 7.1	5.0 (3.0, 9.8)

Data are expressed as mean ± SD (standard deviation) and median (Q1, Q3) (n = 10). Q1 = 25%, Q3 = 75% quantile. LVEF, left ventricular ejection fraction; IVSTd, interventricular septal thickness at end-diastole; LVIDd, left ventricular diastolic internal dimension; LVIDs, left ventricular systolic internal dimension; LVPWTd, left ventricular posterior wall thickness at end-diastole; LVMI, left ventricular mass index (g/m^2^ by body surface area), AV PFV, aortic valve peak flow velocity; AVAI, indexing aortic valve area by body surface area (m^2^); PG, pressure gradient.

### Primary endpoint

**Fig. 1:**
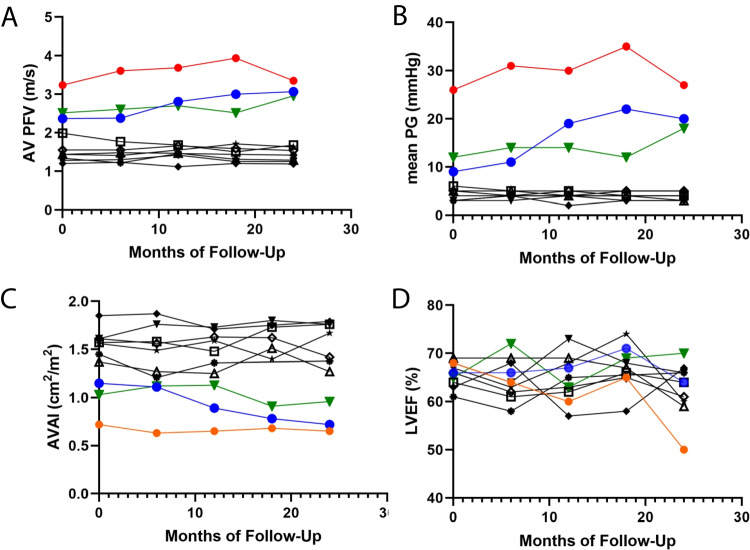
Spaghetti graphs displaying the changes in aortic valve peak flow velocity (AV PFV) **(A)**, mean pressure gradient (PG) **(B)**, aortic valve area index (AVAI) **(C)**, and left ventricular ejection fraction (LVEF) **(D)** over the 24-month follow-up period in 10 osteoporosis patients. In the figure, case 1 is represented by a red circle and line, case 4 by a blue circle and line, and case 7 by a green triangle and line

Figure 1 presents spaghetti graphs illustrating the individual values of AV PFV (A), mean pressure gradient (B), aortic valve area index (C), and left ventricular ejection fraction (D) over the 24-month follow-up period. At baseline, 7 cases had AV PFV <2.0 m/s, while 3 cases had ≥2.0 m/s (n = 2 with mild AS; n = 1 with moderate AS). The cases with AV PFV ≥2 m/s developed moderate AS. Absolute change in AV PFV (m/s) during the first 12 months of follow-up was 0.44, 0.44, and 0.18 for cases 1, 4, and 7, respectively. Over the 24 months of follow-up, these values were 0.11, 0.70, and 0.43 for the same cases, respectively. Case 1 exhibited the highest AV PFV and mean pressure gradient, and the lowest aortic valve area, and ultimately a reduction in left ventricular ejection fraction from 68% to 50% during the course of follow-up. Among the seven cases with AV PFV <2 m/s at baseline, no progression of AS was observed.

### Secondary endpoint

**Fig. 2:**
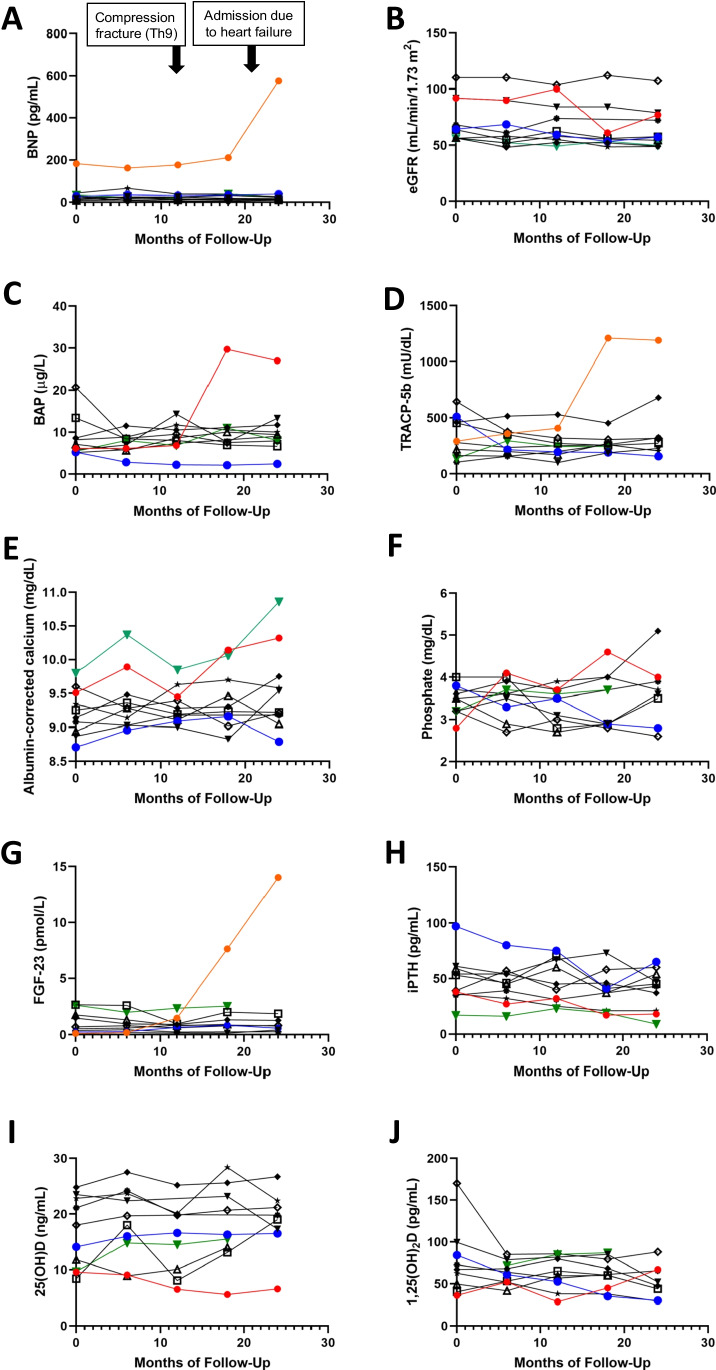
Spaghetti graphs depicting the changes in brain natriuretic peptide (BNP) **(A)**, estimated glomerular filtration rate (eGFR) **(B)**, bone alkaline phosphatase (BAP) **(C)**, tartrate resistance acid phosphatase (TRACP)-5b **(D)**, albumin-corrected calcium **(E)**, phosphate **(F)**, fibroblast growth factor (FGF)-23 **(G)**, intact parathyroid hormone (iPTH) **(H),** 25-hydroxyvitamin D (25(OH)D) **(I)**, and 1,25-dihydroxyvimatin D (1,25(OH)_2_D) **(J)** over the 24-month follow-up period in 10 osteoporosis patients. In the figure, case 1 is represented by a red circle and line, case 4 by a blue circle and line, and case 7 by a green triangle and line. The clinical events of case 1 are indicated at the specified time in panel A

**Fig. 3:**
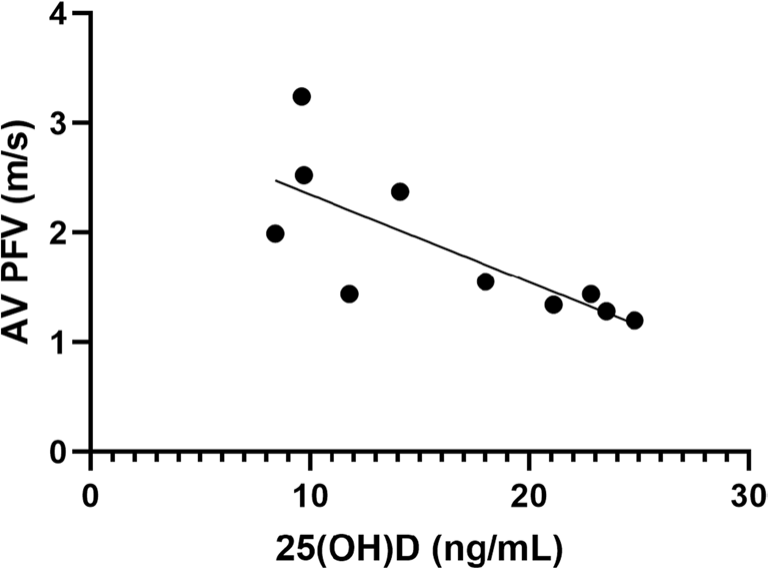
Relationship between aortic valve peak velocity (AV PFV) and 25-hydroxyvitamin D (25(OH)D) levels in 10 osteoporosis patients at baseline. Linear regression was performed to determine the predictive value of 25(OH)D on AV PFV

Figure 2 presents spaghetti graphs displaying individual values of BNP (A), eGFR (B), BAP (C), TRACP-5b (D), albumin-corrected calcium (E), phosphate (F), FGF-23 (G), iPTH (H), 25(OH)D (I), and 1,25(OH)_2_D (J) over the 24-month follow-up period. There were significant variations in terms of AS progression and biomarkers associated with bone turnover among the enrolled patients. Case 1 experienced overt heart failure accompanied by an elevation in BNP levels during the observation periods. This patient exhibited increased FGF-23 levels, followed by elevations in BAP and TRACP-5b levels after switching to daily teriparatide administration. Meanwhile, the plasma 25(OH)D levels consistently decreased over the 24-month period. Figure 3 demonstrates a negative relationship between plasma 25(OH)D levels and AV PFV values at baseline, indicating that higher AV PFV values were associated with decreased plasma 25(OH)D levels (β = -0.756, 95% CI, -0.136 – -0.023, p = 0.011).

## Discussion

This exploratory study provides the first-time demonstration of the temporal changes in aortic valve morphology and parameters of bone and mineral metabolism in osteoporosis patients receiving anti-resorptive therapy such as bisphosphonates or desunomab. Our data provide support for the notion that the pre-existing aortic valve calcification is a predisposing factor for the progression of AS [28, 29]. Additionally, our findings suggest a potential association between low vitamin D status and aortic valve progression. Mineralization plays a central role in the progression of aortic valve calcification at some point [4, 14]. In this study, measurements of BMD, T-score values, BNP, BAP, TRACP-5b, FGF-23, RANKL, OPG, or 1,25(OH)_2_D levels were found to be insensitive in terms of predicting the progression of AS over the 24-month follow-up period (data not shown). Low vitamin D status contributes to bone loss by stimulating PTH production [30]. 25(OH)D levels are measured to assess vitamin D status adequacy [31]. In this study, all patients included exhibited low vitamin D status. Importantly, the severity of AS, as assessed by AV PFV, could be predicted by the levels of 25(OH)D at baseline. Deficient levels of vitamin D have been implicated in stimulating pro-inflammatory factors and pathways, promoting osteogenic differentiation, and enhancing metalloproteinase activity, ultimately leading to vascular calcification [32]. It is postulated that co-existing vitamin D status may determine the extent of aortic valve calcification. The serum level of 1,25(OH)_2_D is tightly regulated by PTH, and it reflects both endogenous production and the effect of activated vitamin D_3_ analogs in this study [16, 33]. The relationship between serum calcium levels and the progression of AS has not been conclusively established [34, 35]. However, calcium supplementation, with or without native vitamin D, has been associated with a higher incidence of future aortic valve replacement in elderly patients with mild to moderate AS [35]. Among the patients in this study, one out of three (case 7) who were taking activated vitamin D_3_ analog experienced AS progression along with an increase in serum calcium levels exceeding 10.5 mg/dL [36]. The decreased iPTH levels and increased 1,25(OH)_2_D levels suggest excessive administration of activated vitamin D_3_ analog. Further research is needed to determine whether activated vitamin D_3_ analogs have an additive risk in AS progression. The natural history of AS typically involves a prolonged asymptomatic period, but symptoms develop, initially presenting as exertional dyspnea, and progressing to heart failure [4]. The elevated levels of BNP and FGF-23 suggest a potential link between osteoporosis and the worsening of heart failure [37, 38]. Furthermore, FGF-23, derived from osteocytes, has been shown to have effects on the deterioration of left ventricular function and the acceleration of valvular calcification [39, 40]. The current report raises the possibility that assessment of aortic valve morphology and circulating 25(OH)D should be performed prior to initiation of osteoporosis treatment. It is plausible that clinicians should consider that osteoporosis patients with mild to moderate AS may experience further progression when they have low vitamin D status. This information can help personalize the follow-up period of echocardiogram and aid in determining the need for valve intervention.

This study has several limitations. Firstly, the sample size was very small, and this may limit the generalizability of the findings. Additionally, there is a possibility of selection bias, which could have influenced the prevalence of AS in the study population. The assessment of AS severity relied on echocardiography, which is subjected to measurement errors, and hemodynamic parameters such as peak velocity, mean pressure gradient, aortic valve area can be influenced by factors like left ventricular function, aortic compliance, and presence of hypertension [41, 42]. Another limitation is that we did not measure bone remodeling markers such as carboxyterminal cross-linked telopeptide type 1 collagen and N-terminal propeptide of type 1 procollagen in this study [43]. Furthermore, the potential contribution of genetic predisposition, such as variation in the vitamin D receptor genotype [44], and variation in response to activated vitamin D_3_ analogs [45], warrant further investigation in relation with AS progression. It is also important to note that the impact of the duration of low vitamin D status prior to enrollment on the progression of valvular calcification remained to be determined. However, it is plausible that sustained low vitamin D status may have contributed to the progression of AS, and investigating whether vitamin D status can be modified as a risk factor presents a challenge.

In conclusion, our findings indicate that low vitamin D status might be a potential risk factor for AS progression.

### Supplementary Information

ESM 1 (PPTX 46 kb)

## Data Availability

The original contributions presented in the study are included in the article/supplementary materials. Further inquiries can be directed to the corresponding author.
