# Supplementary material for: Increasing baseline aortic valve peak flow velocity is associated with progression of aortic valve stenosis in osteoporosis patients—a possible link to low vitamin D status
Source: Arch Osteoporos. 2023 Oct 24;18(1):129. doi: 10.1007/s11657-023-01339-2 (PMC10598115; doi:10.1007/s11657-023-01339-2)
Supplement: Supplementary file 1 — (PPTX 46 kb) [file 11657_2023_1339_MOESM1_ESM.pptx]

## Slide 1
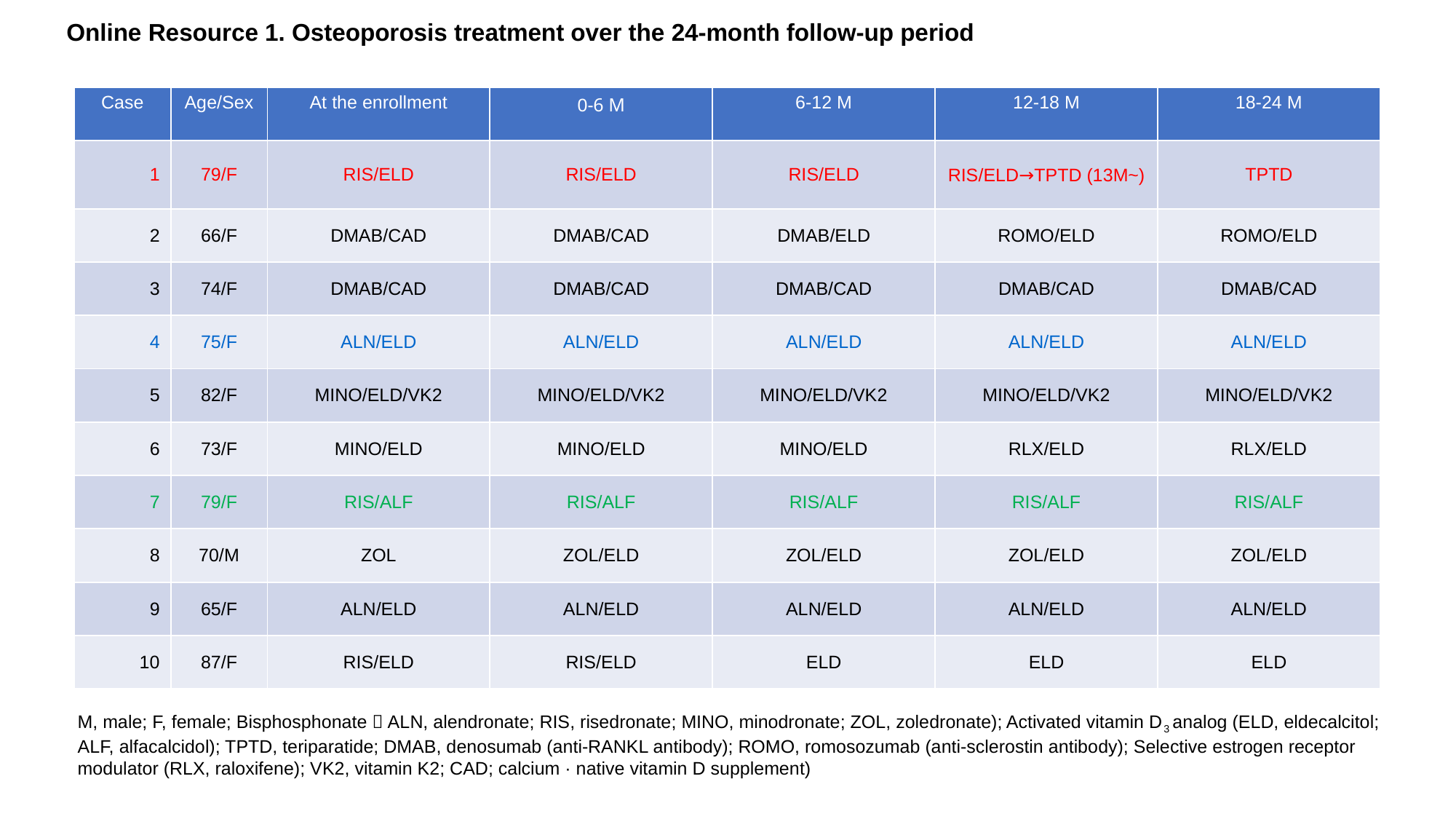

Online Resource 1. Osteoporosis treatment over the 24-month follow-up period
| Case | Age/Sex | At the enrollment | 0­­­-6 M | 6-12 M | 12-18 M | 18-24 M |
| --- | --- | --- | --- | --- | --- | --- |
| 1 | 79/F | RIS/ELD | RIS/ELD | RIS/ELD | RIS/ELD→TPTD (13M~) | TPTD |
| 2 | 66/F | DMAB/CAD | DMAB/CAD | DMAB/ELD | ROMO/ELD | ROMO/ELD |
| 3 | 74/F | DMAB/CAD | DMAB/CAD | DMAB/CAD | DMAB/CAD | DMAB/CAD |
| 4 | 75/F | ALN/ELD | ALN/ELD | ALN/ELD | ALN/ELD | ALN/ELD |
| 5 | 82/F | MINO/ELD/VK2 | MINO/ELD/VK2 | MINO/ELD/VK2 | MINO/ELD/VK2 | MINO/ELD/VK2 |
| 6 | 73/F | MINO/ELD | MINO/ELD | MINO/ELD | RLX/ELD | RLX/ELD |
| 7 | 79/F | RIS/ALF | RIS/ALF | RIS/ALF | RIS/ALF | RIS/ALF |
| 8 | 70/M | ZOL | ZOL/ELD | ZOL/ELD | ZOL/ELD | ZOL/ELD |
| 9 | 65/F | ALN/ELD | ALN/ELD | ALN/ELD | ALN/ELD | ALN/ELD |
| 10 | 87/F | RIS/ELD | RIS/ELD | ELD | ELD | ELD |
M, male; F, female; Bisphosphonate（ALN, alendronate; RIS, risedronate; MINO, minodronate; ZOL, zoledronate); Activated vitamin D3 analog (ELD, eldecalcitol; ALF, alfacalcidol); TPTD, teriparatide; DMAB, denosumab (anti-RANKL antibody); ROMO, romosozumab (anti-sclerostin antibody); Selective estrogen receptor modulator (RLX, raloxifene); VK2, vitamin K2; CAD; calcium · native vitamin D supplement)
